# Photoacoustic imaging of voltage responses beyond the optical diffusion limit

**DOI:** 10.1038/s41598-017-02458-w

**Published:** 2017-05-31

**Authors:** Bin Rao, Ruiying Zhang, Lei Li, Jin-Yu Shao, Lihong V. Wang

**Affiliations:** Biomedical Engineering Department, Washington University of Saint Louis MO, Saint Louis, MO 63130 USA

## Abstract

Non-invasive optical imaging of neuronal voltage response signals in live brains is constrained in depth by the optical diffusion limit, which is due primarily to optical scattering by brain tissues. Although photoacoustic tomography breaks this limit by exciting the targets with diffused photons and detecting the resulting acoustic responses, it has not been demonstrated as a modality for imaging voltage responses. In this communication, we report the first demonstration of photoacoustic voltage response imaging in both *in vitro* HEK-293 cell cultures and *in vivo* mouse brain surfaces. Using spectroscopic photoacoustic tomography at isosbestic wavelengths, we can separate voltage response signals and hemodynamic signals on live brain surfaces. By imaging HEK-293 cell clusters through 4.5 mm thick *ex vivo* rat brain tissue, we demonstrate photoacoustic tomography of cell membrane voltage responses beyond the optical diffusion limit. Although the current voltage dye does not immediately allow *in vivo* deep brain voltage response imaging, we believe our method opens up a feasible technical path for deep brain studies in the future.

## Introduction

A human brain generates thoughts, perceptions, memories, actions, and emotions based on environments and experiences, while defining each individual person. Despite the similarities of human brains, the detailed connections and interactions of brain neuron circuits are uniquely shaped by a person’s life. Understanding the brain will not only significantly advance human knowledge, but also catalyze new treatment modalities for devastating brain diseases such as Parkinson’s disease^1–3^ and Alzheimer’s disease^4, 5^.

To decipher human brains, we need to measure neuronal activity with higher precision, and over much larger spatial and temporal scales^6–9^ than currently achievable. However, current voltage recording tools, including electrodes and optical microscopes, are underdeveloped. Microelectrodes and macro-electrodes are workhorses in recording neural voltage response signals and stimulating neural tissue^10, 11^ in clinics and labs. But they are limited by the small number of practicable simultaneous recording points and they cannot interrogate tiny neuronal circuit components such as dendrites and axon terminals. More recently, non-invasive optical methods for recording activity in large numbers of neurons have been significantly improved^12, 13^. Optical, chemical, and genetic sensors have enabled scientists to study microcircuits and understand sub-cellular neurochemical dynamics in dendrites and axons^12–17^. Despite recent innovations in optical sensors^18, 19^ and non-invasive optical imaging instruments, much brain research with non-invasive optical methods is confined to the neuronal circuits of the top layer of the brain^20^. *In vivo* imaging of deep brain neuronal tissue and neuronal activity remains an unmet challenge.

Photoacoustic tomography^21–25^ (PAT) is a hybrid optical imaging method that detects acoustic responses excited by laser pulses of both ballistic and diffused photons and achieves high-resolution optical-contrast tomography at imaging depths beyond the optical diffusion limit^26^. Specifically, optical absorbers at all imaging depths absorb pulsed laser energy and radiate photoacoustic waves that are detected by acoustic sensors for rendering three-dimensional images of the optical absorbers. Although PAT is a versatile optical imaging modality, its potential in imaging voltage responses of neuronal activities has never been explored.

In this communication, for the first time, we demonstrate photoacoustic voltage response imaging contrast by using a non-radiative voltage sensor (dipicrylamine, or DPA for short). By using spectroscopic photoacoustic tomography at isosbestic wavelengths, we can successfully separate the voltage response signals from the hemodynamic signals on live brain surfaces. We demonstrate the photoacoustic detection of HEK-293 cell membrane voltages through 4.5 mm thick *ex vivo* rat brain tissue. Although the current voltage dye (DPA) does not immediately allow *in vivo* deep brain voltage response imaging, we believe our method opens up a feasible technical path for deep brain studies in the future.

## Results and Discussion

### Photoacoustic voltage response contrast

**Figure 1:**
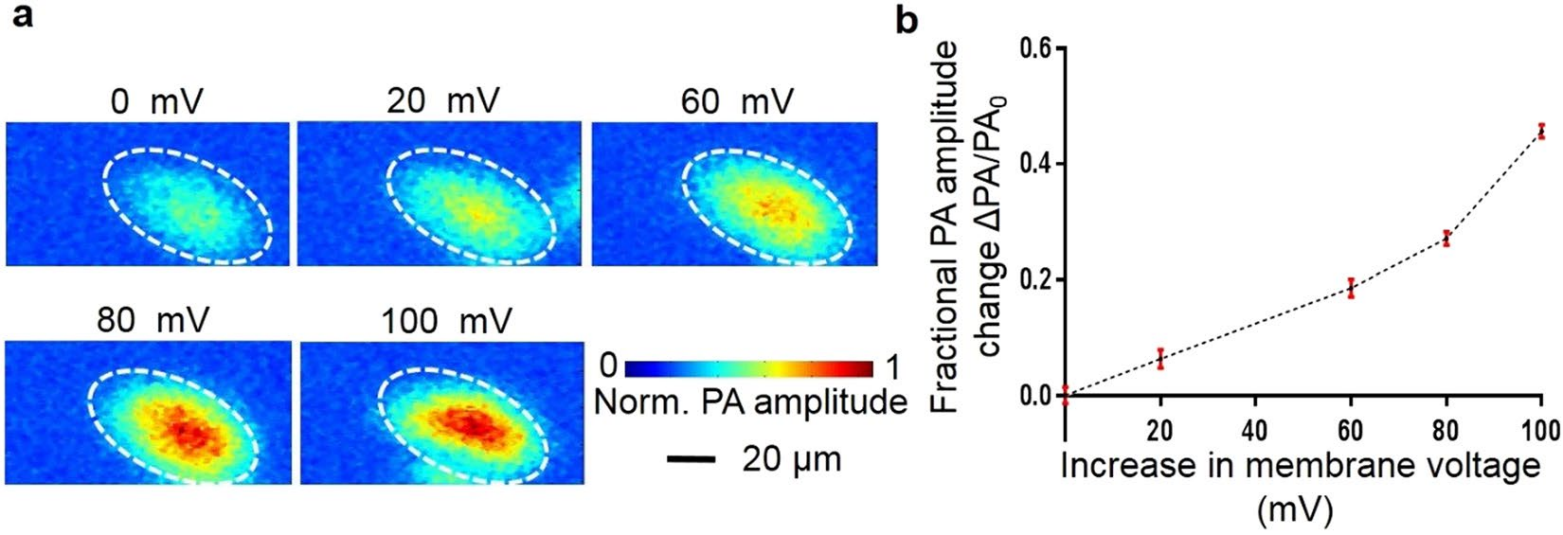
(**a**) Photoacoustic maximum-amplitude projection images of HEK-293 cells demonstrate photoacoustic signal changes due to cell membrane voltage changes. (**b**) Fractional photoacoustic signal change versus cell membrane resting voltage changes.

Hydrophobic anions, such as DPA and sodium tetraphenylborate (TphB), have extremely strong adsorption to plasma membranes, and their adsorption and transport mechanism in the lipid bilayer membrane have been well studied^27, 28^. So far, DPA has been found to induce voltage-dependent membrane capacitance in the squid giant axon^29^ and to report neuron cell membrane potentials^30–35^ in fluorescence imaging. We wondered if it could be used as a voltage sensor for photoacoustic tomography. Thus, we conducted an *in vitro* HEK-293 cell imaging experiment with a transmission mode optical-resolution photoacoustic microscope (Figure s2), as detailed in the supplementary information (SI), section II. Figure 1a shows how photoacoustic signals change in response to cell membrane potential changes. The dashed ellipse in Fig. 1a identifies a HEK-293 cell cluster. The larger the HEK-293 cell membrane resting potential change, the larger the photoacoustic signal change. Figure 1b quantifies the fractional photoacoustic signal changes due to cell membrane voltage changes estimated from the Nernst equation. First, we calculate the averaged photoacoustic amplitude (PA) signal for cells within the dashed ellipse for each image. Second, we compute the fractional PA signal change by normalizing the PA signal change by the initial PA signal under the initial membrane potential. A fractional photoacoustic signal increase greater than 40% was recorded for a 100 mV membrane resting potential change. Because the Nernst equation overestimates voltage change^36^, an even larger fractional photoacoustic signal change was expected for a given membrane resting potential change. It is worth noting that we might have a slightly different PA voltage response curve in Fig. 1b if the more accurate Goldman equation is used.

Additionally, we use di-4-ANEPPS, a fluorescence voltage-sensitive dye (D1199, Molecule Probes) and an inverted Olympus IX61 microscope to experimentally verify the HEK-293 cell membrane resting potential change in response to potassium ion concentration changes in the cell culture. A fluorescence voltage response curve (Figure s4b) similar to Fig. 1b is observed (SI, section IV). Further quantitative voltage imaging is beyond the scope of the current study.

The PA signal can be written as p = αξΓmμ*F*, where p denotes the PA signal, α is a spatially dependent factor, ξ is a coefficient representing the distortion and attenuation of the photoacoustic signal, Γ denotes the Grϋneisen parameter of the medium, m denotes the molar concentration of DPA, μ denotes the molar optical absorption coefficient of DPA, and *F* denotes the local optical fluence in J/m^2^. Because α, ξ, Γ, and *F* are parameters not affected by plasma membrane voltage, the PA voltage response contrast must originate from either the molar concentration (m) or the molar optical absorption coefficient (μ). In the case of the voltage-dependent molar optical absorption coefficient (μ), a voltage-dependent optical absorption spectrum is expected. To investigate whether the voltage-sensitive mechanism is due to the molar optical absorption coefficient (μ), we performed photoacoustic spectroscopy of the DPA-stained HEK-293 cell membrane under two different resting potentials. We found that the normalized photoacoustic absorption spectrum did not change with membrane voltage (detailed in the SI, section V), which leaves, as the only explanation, that the molar concentration (m) of DPA in lipid membrane is voltage-dependent. This voltage sensitive mechanism is very close to repartitioning^18^, a fluorescent voltage sensitive scheme that solely involves dye molecules moving in and out of the lipid membrane with voltage changes. The only difference is that the dye molecules involved here are non-fluorescent.

To further verify the hypothesis, we conducted another experiment with spectrophotometry instead of photoacoustics. In this experiment, we stained HEK-293 cells with 5 µM DPA solutions with different K^+^ concentrations. The total number of DPA molecules, including DPA adsorbed onto plasma membrane and DPA in solution, remained constant. By quantitatively measuring the DPA molecules left in the solution with spectrophotometry, we calculated the DPA molecules adsorbed to the membrane under different membrane voltages. The spectrophotometric results are detailed in the SI, section VI. Both spectrophotometric results and photoacoustic results agreed well. Thus, the spectrophotometry confirmed repartitioning, i.e., that more DPA molecules are adsorbed to the cell membrane when the resting potential change is higher, which leads to stronger photoacoustic signals. We conclude that repartitioning of DPA molecules is the voltage sensitive mechanism behind the PA voltage response contrast. This type of voltage sensitive mechanism is considered not fast enough for imaging dynamic action potentials of neurons. The frequency response of the DPA dye could be modeled as the low-pass filtered frequency response of an ordinary voltage dye.

### *In vivo* mouse brain surface voltage response to electrical stimulation

Although DPA voltage dye is slow, we still could use it to record voltage responses in mouse brains *in vivo*. One obstacle for PA voltage response imaging is the interference of hemoglobin in blood. Deán-Ben XL *et al*. reported *in vivo* PAT imaging of GCaMP5G (a genetic calcium indicator) zebrafish^37^. However, the contributions of hemoglobin and GCaMP5G in the PA signal were not separated because hemoglobin signal could be very similar to the calcium signal due to the well-known neuronal-vascular coupling effect. Here, we considered a simplified case where the imaging target is the DPA stained dura layer immediately beneath the mouse skull instead of neuron circuits inside the live brain. The laser fluence in the imaging voxel, which includes both the dura and cortex tissue, is a constant after normalization against the photodiode recording of laser pulse energy. For this simplified case, a spectroscopic method provides exact solutions for both the hemodynamic signal, and voltage response signal, as detailed in the Methods section. The analysis of calculation error presented in the SI, section X, shows that the calculation errors are less than 1.5 times the noise. This spectroscopic voltage response imaging method is very similar to the spectroscopic oxygen saturation imaging method^38, 39^ that was first verified with *in vitro* phantoms and then applied to *in vivo* applications. By measuring the DPA and blood concentrations in DPA and blood mixtures, we verified that our spectroscopic method can accurately resolve the PA signal of DPA dye from different mixtures (SI, section VII). The voltage response signal is a low-pass filtered local field potential signal, which has a high similarity within a relatively large area^40^. Thus, we expect that motion artifacts caused by respiration have minor effect on the measured voltage response signal.

As we know, electrical stimulation can change the neuronal membrane voltage and trigger a neuronal response to extracellular electrical currents near the neuron^41–43^. This stimulation approach has been used to identify the motor cortex, map neural connections between brain regions, modulate attention, increase the speed of learning, identify neural subtypes, record movement sequences, study somatosensory perception, and introduce signals directly into the brain in brain-machine interface models. It was shown that micro-electrical stimulation sparsely activates neurons around the electrode up to millimeters away from the point of stimulation^41^. Although we have a limited understanding of the effects of electrical stimulation on individual neurons, it serves well for our purpose of voltage response signal imaging. Animal preparation is detailed in SI, section I. DPA dye was applied outside the intact dura matter. The depth-resolved photoacoustic microscopy image indicated that photoacoustic signals come from the surface of the brain, including micro-vessels within the M-mode imaging voxel volume, where the laser fluence is a constant after normalization against the photodiode recording of the laser pulse energy. The estimated $\frac{P{A}_{Hb}}{P{A}_{DPA}}$ at 500 nm varies from 3.3 to 6.6 in the cranial window (SI, section XI). The fact that PA signals from DPA stained tissue are not overwhelmed by the hemoglobin signals at 500 nm allows the separation of the voltage response signal from the hemoglobin signal.

**Figure 2:**
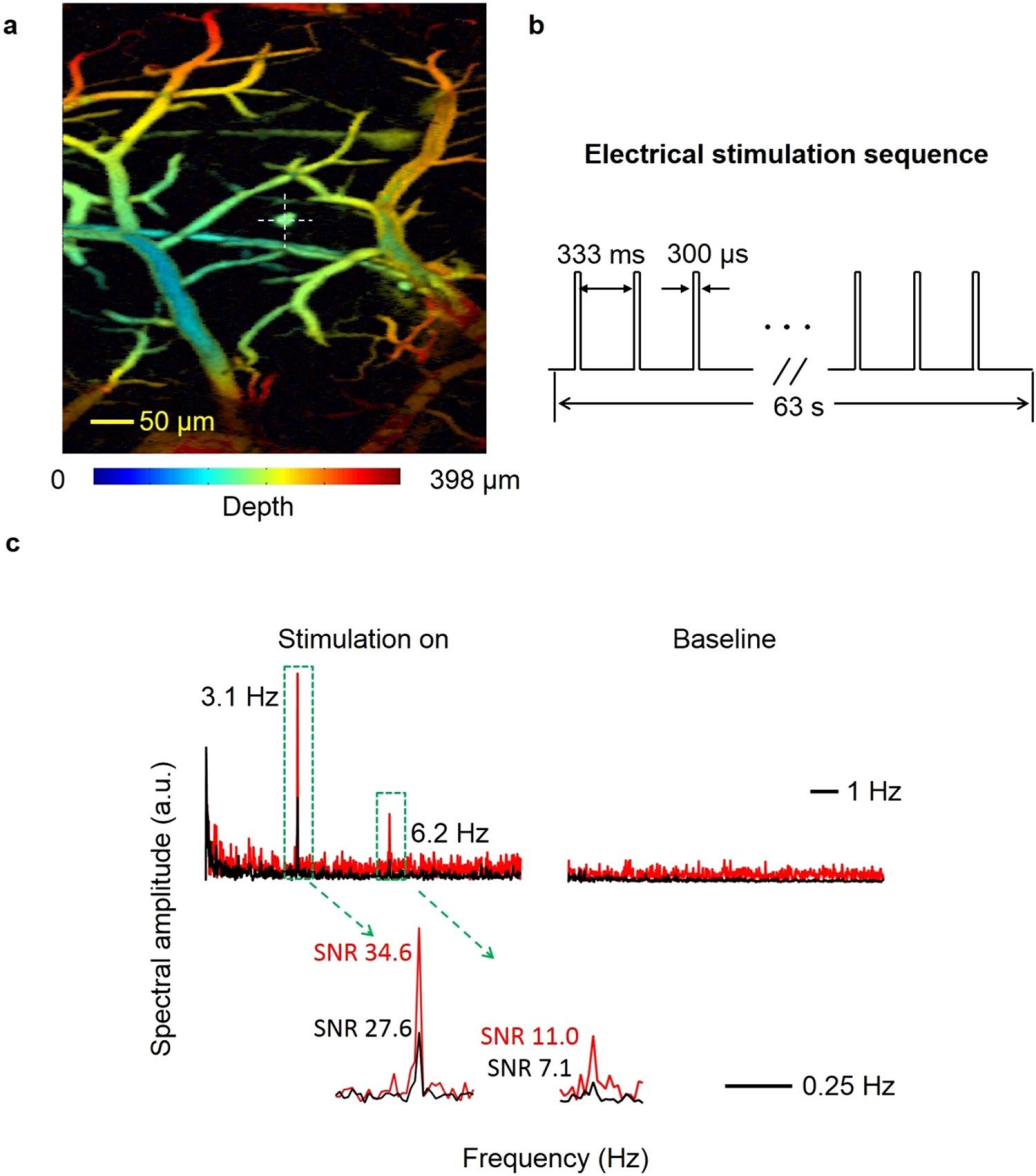
*In vivo* mouse brain response before and during electrical stimulation at a minimally vascularized point. (**a**) The exact M-mode imaging location is identified by the yellow cross in the depth-coded maximum amplitude projection image of the DPA-stained mouse cranial window. (**b**) A 63 second stimulation sequence comprises 189 electrical stimulation pulses with a pulse width of 300 μs and a pulse period of 333 ms. (**c**) Baseline (before electrical stimulation) and response (during stimulation) of voltage and hemodynamic signals in the frequency domain. Both the voltage response signal (red) and hemodynamic response signal (black) have observable frequency components at 3.1 Hz and 6.2 Hz. The SNRs of the voltage and hemodynamic response peaks are respectively labeled with red and black text.

Using a reflection-mode optical-resolution photoacoustic microscope (OR-PAM) detailed in SI, section III, we recorded *in vivo* mouse brain responses before and during electrical stimulation at a point with minimum vasculature. In Fig. 2a, the exact M-mode imaging location is identified by the yellow cross in the depth-coded maximum amplitude projection image of the DPA-stained mouse cranial window. Figure 2b shows a 63-second electrical stimulation sequence that comprises 189 electrical stimulation pulses with a pulse width of 300 μs and a pulse period of 333 milliseconds. There are no observable features in either the baseline (before electrical stimulation) or response (during stimulation). We analyzed the baseline and response of the voltage and hemodynamic signals in the frequency domain. Compared to the noise-like baseline signals, both the voltage response signal (red) and hemodynamic response signal (black) have observable frequency components at 3.1 Hz and 6.2 Hz, as shown in Fig. 2c. The signal-to-noise ratios (SNR) of the voltage and hemodynamic response peaks are labeled with red and black text, respectively, next to the peaks. Because the voltage response signal (red trace) is stronger than hemodynamic signal for both frequency components, the voltage response is dominant at this minimally vascularized M-mode imaging point. The SI, section XII, shows that the electrical stimulation pulse train has frequency components only at k × 3 Hz (k is an integer). If we model the voltage response of DPA dye on the dura layer as a linear low pass filter for all frequency components less than 8 Hz, we end up with two frequency components at 3 Hz and 6 Hz. Our analysis based on the model agrees with the observed PA voltage response peaks at 3.1 Hz and 6.2 Hz. The experimental results have clearly shown that DPA is a slow dye, which is not suitable for further dynamic action potential imaging experiments.

Additionally, we used di-4-ANEPPS dye and an upright Olympus BX61W1 microscope to experimentally verify the *in vivo* mouse brain surface voltage response to electrical stimulation. Similar to previous findings, two peaks at 3.2 Hz and 6.1 Hz in the frequency domain were observed (SI, section VIII).

### *In vivo* mouse brain voltage response signal imaging before and during 4-aminopyridine induced epilepsy

The drug 4-aminopyridine (4-AP) was used to induce epileptic seizure in various models of epileptic seizure^44–48^. Epileptiform activity has been extensively studied using different optical imaging methods^49–56^. Among these, photoacoustic tomography has the potential to record deep brain voltage response signals and hemodynamic signals in parallel and to become a new tool for understanding the induction, maintenance, and propagation of seizure discharges. Here, in an animal model, we demonstrated the recording of both neuronal and hemodynamic responses to epileptic seizure induced by 4-AP. Animal preparation is detailed in SI, section I. The same reflection-mode OR-PAM was used for this experiment.

**Figure 3:**
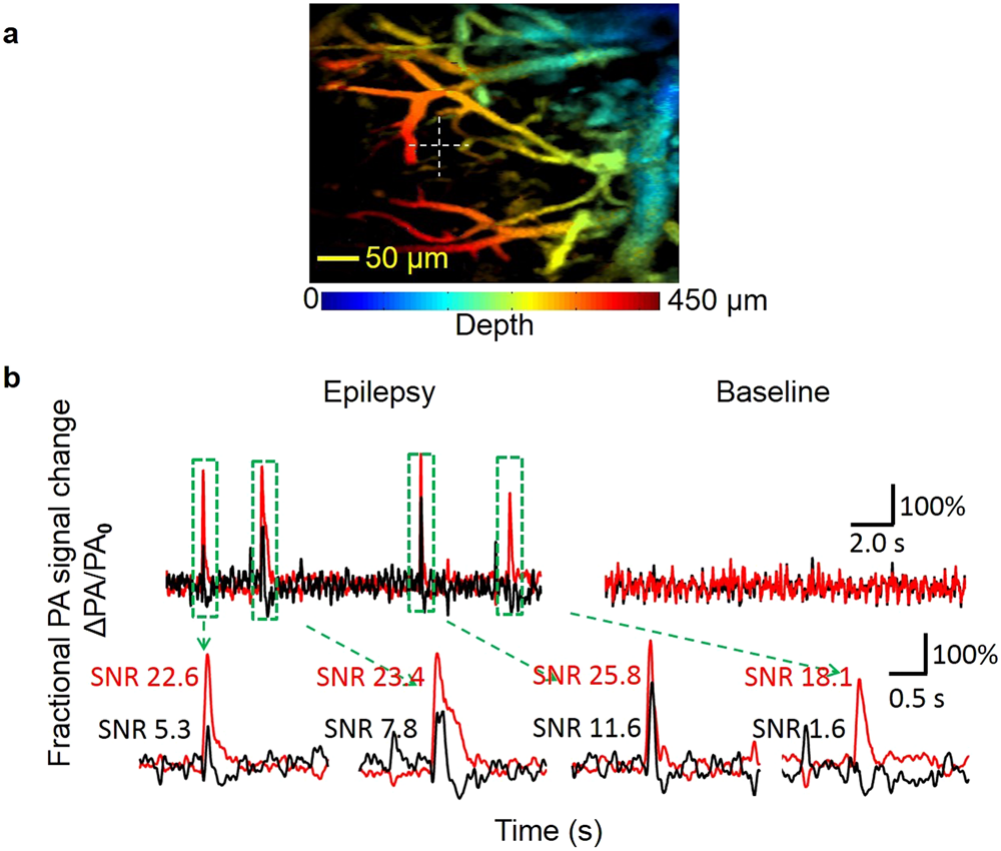
*In vivo* mouse brain response before and during 4-Aminopyridine (4-AP) induced epilepsy. (**a**) The white cross identifies the M-mode recording point on the depth-encoded maximum amplitude projection image of the mouse brain through the cranial window. The color represents depth. (**b**) Fractional voltage and fractional hemodynamic signal changes in the time domain for the baseline (before 4-AP stimulation) and response (during 4-AP induced epilepsy). The SNRs of the voltage and hemodynamic response peaks are labeled with red and black text. The fractional voltage response signal change shows stronger responses (spike amplitudes) than the fractional hemodynamic signal change.

Figure 3a shows a maximum amplitude projection image of a mouse brain through a skull window after staining with DPA. The white cross identifies the location of M-mode imaging. Figure 3b shows the calculated fractional photoacoustic voltage response signal change (red trace) and the fractional hemodynamic signal change (black trace) before and after 4-AP induced epilepsy. Before the induction of epilepsy, both the fractional voltage response signal change and the fractional hemodynamic signal change show no observable events. However, the induced epilepsy after the injection of 4-AP within the skull window generated significant spikes at the same M-mode imaging location. We recorded a fractional photoacoustic voltage response signal change of more than 300% and a fractional photoacoustic hemodynamic signal change of more than 200%. We hypothesize that the significant voltage response signal change could be due to synchronized neuronal firing during 4-AP induced epilepsy and that the significant hemodynamic signal change could be related to the recruitment of capillaries during epilepsy and the neuronal-vascular coupling. We note that the voltage-sensitive mechanism of DPA dye reflects its voltage-dependent adsorption to the lipid membrane. This mechanism is considered too slow to catch the single action potentials fired by the epileptic neurons. The calculated fractional voltage response signal change that has been low-pass filtered by the DPA dye could show only the entire epileptic spike envelopes. Both the fractional voltage response signal change (red) and the fractional hemodynamic response signal change (black) show spikes that are not necessarily always synchronous. Over all, at the recording point, the fractional voltage response signal change shows stronger responses than the fractional hemodynamic signal change.

### Imaging HEK-293 cell clusters with different resting potentials through thick *ex vivo* brain tissue

In the above two *in vivo* imaging experiments, we demonstrated PA imaging of voltage responses as a function of the brain surface voltage. In addition to the easy dye delivery and robustness against respiration-caused motion artifacts, both animal models allow exact solutions of the voltage response signal and the hemoglobin signal. *In vivo* deep brain voltage response imaging remains very challenging without a long-wavelength voltage sensor whose optical absorption spectrum can be separated from that of hemoglobin. However, the DPA voltage dye still allows us to demonstrate *in vitro* PA voltage response imaging at depths beyond the optical diffusion limit^24^. A photoacoustic computed tomography system (Figure s9, SI, section IX) was used for this demonstration experiment.

**Figure 4:**
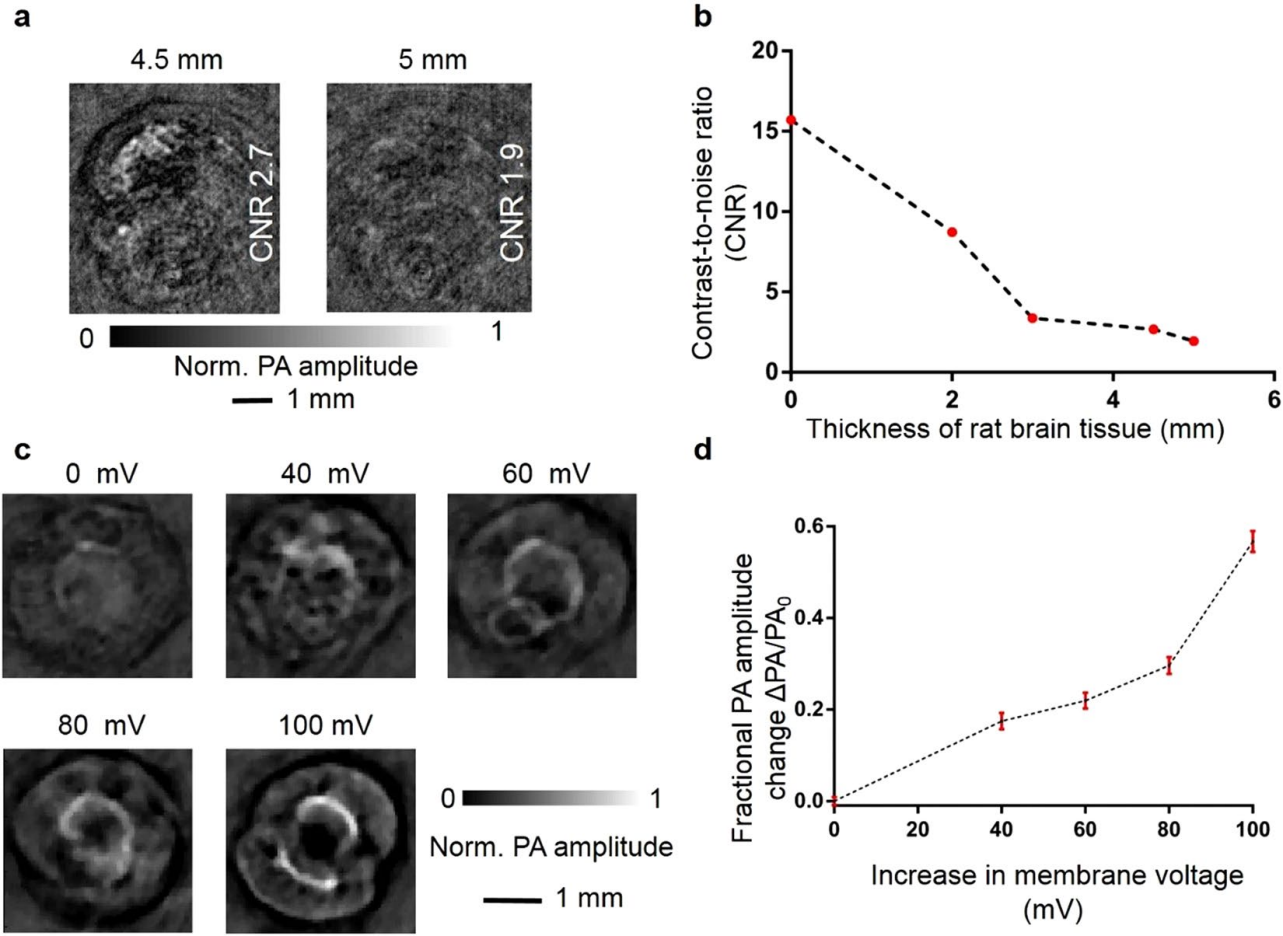
Imaging HEK-293 cell clusters with different resting potentials through different thicknesses of *ex vivo* brain tissue. (**a**) HEK-293 cell cluster PACT images through 4.5 mm and 5.0 mm thick brain tissue. (**b**) The contrast-to-noise ratios of the HEK-293 cell cluster images acquired through 0.0 mm, 2.0 mm, 3.0 mm, 4.5 mm, and 5.0 mm thick brain tissue decrease as the brain tissue thickness increases. (**c**) Photoacoustic images of HEK-293 cell clusters acquired through 4.5 mm thick brain tissue in response to cell membrane voltage changes. (**d**) Fractional photoacoustic signal change versus cell membrane voltage change.

As described in the methods section, we first imaged HEK-293 cell clusters through different thicknesses of rat brain tissue. Figure 4(a) shows PACT images of HEK-293 cell clusters acquired through 4.5 mm and 5.0 mm thick rat brain tissues. The contrast-to-noise ratios (CNR) of the cell cluster images acquired through 0.0 mm, 2.0 mm, 3.0 mm, 4.5 mm, and 5.0 mm thicknesses decrease with increasing thickness of brain tissue, as shown by Fig. 4(b). The calculation of CNR is detailed in supplementary info section IX. We define the cell cluster visibility threshold as CNR of at least 2. Consequently, the HEK-293 cell clusters remain visible through 4.5 mm thick rat brain tissue and become invisible through 5 mm thick rat brain tissue.

Second, we imaged HEK-293 cell clusters in response to different cell membrane voltage changes through 4.5 mm thick rat brain tissue. In Fig. 4(c), stronger photoacoustic signals were observed due to larger cell membrane voltage changes. The fractional photoacoustic amplitude change versus voltage change is plotted in Fig. 4(d). There is more than a 60% fractional PA amplitude change with 100 mV of cell membrane voltage change, which might have been caused by the longer staining time when cell clusters were bathed in DPA solutions during the imaging procedures.

Our results are still limited by the sensitivity and density of the ultrasonic transducer array device. High-sensitivity, high-frequency optical sensors with excitation wavelengths outside of the hemoglobin absorption spectrum will maximize the achievable imaging depth of PACT imaging.

## Conclusions

We report the first demonstration of photoacoustic voltage response imaging in both *in vitro* HEK-293 cell cultures and *in vivo* mouse brain surfaces. Using spectroscopic photoacoustic tomography at isosbestic wavelengths, we can separate voltage response signals and hemodynamic signals on live brain surfaces. By imaging HEK-293 cell clusters through 4.5 mm thick *ex vivo* rat brain tissue, we demonstrate photoacoustic tomography of HEK-293 cell membrane voltage responses beyond the optical diffusion limit. Although the current voltage dye does not allow *in vivo* deep brain voltage response imaging immediately, we believe our method opens up a feasible technical path for deep brain studies in the future.

## Methods

### Principle of separating voltage response signals from hemodynamic signals in live brains

The amplitudes of brain photoacoustic signals from hemoglobin and DPA dye excited by a nanosecond laser pulse are written as

1 $${{\rm{p}}}_{Hb}={{\rm{\alpha }}{\rm{\xi }}{\rm{\Gamma }}{\rm{m}}}_{Hb}{{\rm{\mu }}}_{Hb}F$$

2 $${{\rm{p}}}_{DPA}={{\rm{\alpha }}{\rm{\xi }}{\rm{\Gamma }}{\rm{m}}}_{DPA}{{\rm{\mu }}}_{DPA}F,$$

where the subscripts Hb and DPA denote hemoglobin and DPA respectively, p denotes the amplitude of the photoacoustic signal, α is a spatially dependent factor, ξ is a coefficient representing distortion and attenuation of the photoacoustic signal, Γ denotes the Grϋneisen parameter of the medium, m denotes the molar concentration, μ denotes the molar optical absorption coefficient of hemoglobin, and *F* denotes the local optical fluence in J/m^2^. The molar optical absorption coefficients of hemoglobin and DPA dye are known. The molar concentrations of hemoglobin and DPA are unknown. In order to separate DPA signals from hemodynamic signals, two measurements are required to solve the equations of the two absorbers.

For our two measurements, we selected two isosbestic wavelengths of hemoglobin (500 nm and 570 nm), where oxygenated and deoxygenated hemoglobin molecules have the same molar absorption coefficient values. Hence, the photoacoustic signals are dependent on the total concentration of hemoglobin only and are independent of the oxygen saturation of hemoglobin (i.e., the ratio of the concentration of oxygenated hemoglobin to the concentration of total hemoglobin). The laser fluence *F* was considered as constant after the calibration procedure described later in the *in vivo* experiments. We define ${{\rm{M}}}_{Hb}={{\rm{\alpha }}{\rm{\xi }}{\rm{\Gamma }}m}_{Hb}F$ and ${{\rm{M}}}_{DPA}={{\rm{\alpha }}{\rm{\xi }}{\rm{\Gamma }}{\rm{m}}}_{DPA}F$ as the relative measurements of the concentrations. For the first measurement at 500 nm and the second measurement at 570 nm, we have the following:

3 $${{\rm{\mu }}}_{Hb500}{{\rm{M}}}_{Hb}+{{\rm{\mu }}}_{DPA500}{{\rm{M}}}_{DPA}={{\rm{p}}}_{500}$$

4 $${{\rm{\mu }}}_{Hb570}{{\rm{M}}}_{Hb}+{{\rm{\mu }}}_{DPA570}{{\rm{M}}}_{DPA}={{\rm{p}}}_{570}.$$

Here, the subscripts 500 and 570 indicate the corresponding wavelengths in nm. The relative DPA and hemoglobin concentrations are computed as follows:

5 $${{\rm{M}}}_{DPA}={({\rm{p}}}_{570}-\frac{{{\rm{\mu }}}_{Hb570}}{{{\rm{\mu }}}_{Hb500}}{{\rm{p}}}_{500})/({{\rm{\mu }}}_{DPA570}-\frac{{{\rm{\mu }}}_{Hb570}}{{{\rm{\mu }}}_{Hb500}}{{\rm{\mu }}}_{DPA500})$$

6 $${{\rm{M}}}_{Hb}={({\rm{p}}}_{500}-\frac{{{\rm{\mu }}}_{DPA500}}{{{\rm{\mu }}}_{DPA570}}{{\rm{p}}}_{570})/({{\rm{\mu }}}_{Hb500}-\frac{{{\rm{\mu }}}_{DPA500}}{{{\rm{\mu }}}_{DPA570}}{{\rm{\mu }}}_{Hb570}).$$

The method for separating DPA and hemoglobin signals with the above equations was successfully tested by measuring phantoms with preset concentrations, as detailed in the supplementary information.

### Method for recording DPA responses to different cell membrane resting potentials

Cell membrane resting potentials were adjusted by changing the extracellular potassium ion concentration. The membrane potential change (Δ*E*) is roughly estimated by

7 $${\rm{\Delta }}E=(\frac{RT}{F})\mathrm{ln}(\frac{{K}_{a}}{{K}_{i}}),$$

where *R* is the universal gas constant, *T* is the absolute temperature, *F* is the Faraday constant, and *K*_*a*_ and *K*_*i*_ are the adjusted and initial potassium concentrations. Equation (7) is derived from the Nernst equation, with the assumptions that the internal ion concentrations remain constant during the short exposure to increased extracellular potassium ion concentration and that the impact of the Na^+^ channel is disregarded^34^.

The stock DPA dye solution is 2 mM DPA (Biotium) in DMSO. The DPA stock solution was diluted to 5 μM for *in vitro* cell culture experiments. The initial extracellular medium was made by adding 20 mM HEPES and 5 μM DPA to Hanks’ Balanced Salt Solution (1.3 mM CaCl_2_, 5.4 mM KCl, 0.4 mM KH_2_PO_4_, 0.5 mM MgCl_2_, 0.4 mM MgSO_4_, 136.9 mM NaCl, 0.3 mM Na_2_HPO_4_, 4.2 mM NaHCO_3_, and 5.5 mM glucose), and adjusting its pH to 7.4. Four adjusted extracellular media with potassium concentrations of 12.7 mM, 61.4 mM, 134.8 mM, and 296.0 mM were prepared by adding more KCl to the initial extracellular medium in order to adjust the cell membrane resting potentials by 20 mV, 60 mV, 80 mV, and 100 mV, according to equation (7).

Human embryonic kidney 293 (HEK-293) cells were cultured in DMEM (Invitrogen) supplemented with 10% fetal bovine serum and incubated at 37 °C with 5% CO_2_. For photoacoustic microscopy, HEK-293 cells were plated into a 35 mm glass-bottom petri dish (P35GCOL-0-14-C, MatTek) one day before the experiment. Prior to PAM imaging, 5 μM DPA was added to HEK-293 cell culture medium (DMEM) and HEK-293 cells were stained for 40 minutes. Baseline PAM images corresponding to a 0 mV cell membrane resting potential change were acquired with the initial extracellular medium. Then the medium was sequentially replaced by extracellular media with potassium concentrations of 12.7 mM, 61.4 mM, 134.8 mM, and 296 mM, and immediately imaged by a transmission-mode photoacoustic microscope (see supplementary information) after each replacement. Before each replacement, we reset the DPA concentrations stained on the HEK-293 cells by replacing the old medium with Hanks’ Balanced Salt Solution (no DPA). A waiting time of 5–15 minutes, and occasionally multiple resets, were sometimes required for the photoacoustic signals from the HEK-293 cells to subside to the baseline. The means and standard errors of photoacoustic signal amplitudes from the HEK-293 cells corresponding to five different resting potentials were calculated and analyzed.

### Method of recording *in vivo* mouse brain response to electrical stimulation

We constructed a PAM for *in vivo* mouse brain imaging. As shown in Figure s3, the excitation light sources are (1) an OPO laser system (NT242-SH, Ekspla) and (2) a dye laser (Credo, Sirah) tuned to 570 nm and pumped by a 532 nm Nd:YLF laser (INNOSLAB, Edgewave). The 570 nm laser pulse is delayed by 1 µs relative to the 500 nm laser pulse. A dichroic mirror combines the 500 nm and 570 nm laser beams. To calibrate the fluctuations of laser pulse energy, a beam splitter splits 10% of the laser energies to a transmission-mode optical-resolution photoacoustic microscope that uses a black tape as sample. The remaining 90% of the laser energy is delivered to a miniature photoacoustic imaging probe comprising an achromatic lens (AC064-013-A, Thorlabs) and a customized ring-shaped focused ultrasonic transducer. The mechanical design of the miniature photoacoustic imaging probe allows adjusting the ring transducer for confocal optical and acoustic foci. During imaging, the ring transducer is immersed in the water tank, with a plastic membrane as its bottom. Electrical stimulation electrodes (MX216FW, FHC) are inserted around the edge of the cranial window. Stimulation pulses are sent by an electrical stimulator (Micro-stimu III, World Precision Instruments).

The animal preparation is detailed in the supplementary material. All experimental procedures were carried out in conformity with laboratory animal protocols approved by the Animal Studies Committee at Washington University in St. Louis. For *in vivo* mouse brain experiments, we diluted the stock DPA dye solution of 2 mM DPA (Biotium) in DMSO to 20 μM. Before photoacoustic imaging, 20 µM DPA was applied to the cranial window for 30 minutes of bath staining. For *in vivo* imaging, acoustic gels were placed between the plastic membrane of the water tank and the mouse brain. Initial 3D mouse brain imaging with the OPO laser tuned to 532 nm allowed us to select an M-mode imaging point that appeared to have minimum vasculature. To acquire baseline photoacoustic signals without stimulation, we performed M-mode recordings with dual wavelengths at the selected point. After that, M-mode recordings were collected at the same point during electrical stimulation, and then the photoacoustic responses from hemoglobin and DPA were calculated from equations (5) and (6).

### Method of recording *in vivo* mouse brain response to epilepsy induced by 4-Aminopyridine

We used the same PAM setup to image the mouse brain *in vivo* in response to epilepsy induced by 4-aminopyridine (4-AP). Similar to the method used in the electrical stimulation model, a cranial window was created in the mouse skull and 20 µM DPA was applied to the cranial window for 30 minutes of bath staining. After raster-scan imaging the mouse brain through the cranial window, we selected a point without noticeable interference from blood vessels. A baseline M-mode recording with dual wavelengths was obtained at that point, then 50 µL of 4-AP was injected through the cranial window into the brain cortex to induce focal seizure. After the injection, a raster-scanned image was acquired again to locate the M-mode imaging point. We waited until a visually observable seizure response occurred, and started M-mode recordings of the same location with dual wavelengths. Last, the photoacoustic responses from the hemoglobin and DPA were calculated from equations (5) and (6).

### Method of imaging HEK-293 cell clusters with different resting potentials through thick *ex vivo* brain tissue

We used a photoacoustic computed tomography (PACT) system (supplemental info section VII) to demonstrate that PACT can potentially image deep brain voltage responses. To mimic imaging through different thicknesses of brain tissue, a whole brain was harvested from a sacrificed rat (Sprague Dawley®, Charles River). The whole rat brain was embedded in agarose before being cut into slices either 1 mm or 0.5 mm thick by a vibrating blade microtome (Leica VT 1200 S). Only central slices with relatively large areas were used to construct *ex vivo* brain tissue layers between 2 mm to 5 mm thick. HEK-293 cells that were initially grown in a T-75 flask were washed off with trypsin, immersed in Dulbecco’s Modified Eagle’s Medium (with no phenol red), stained with 5 μM DPA, incubated for 20 minutes in a CO_2_ incubator (37 °C, 5% CO_2_), and distributed among six 15 ml agarose tubes. The HEK-293 cell clusters in the first agarose tube were imaged through different thickness of brain tissue (0.0 mm, 2.0 mm, 3.0 mm, 4.5 mm, and 5.0 mm) in order to determine the maximum thickness of brain tissue through which HEK-293 cell clusters remained visible. The extracellular potassium concentrations in five other agarose tubes were adjusted to achieve 0 mV, 40 mV, 60 mV, 80 mV, and 100 mV of cell membrane voltage changes. HEK-293 cell clusters were then imaged through brain tissue of the determined maximum thickness.

## Electronic supplementary material

Supplementary info of the manuscript
