# Supplementary material for: Photoacoustic imaging of voltage responses beyond the optical diffusion limit
Source: Sci Rep. 2017 May 31;7:2560. doi: 10.1038/s41598-017-02458-w (PMC5451395; doi:10.1038/s41598-017-02458-w)
Supplement: Supplementary file 1 — Supplementary info of the manuscript [file 41598_2017_2458_MOESM1_ESM.pdf]

Photoacoustic imaging of voltage responses beyond the optical diffusion limit

Authors: Bin Rao+, Ruiying Zhang+, Lei Li, Jin-Yu Shao, Lihong V. Wang*

+These authors contributed equally to this work

*Corresponding author

Affiliations:

Biomedical Engineering Department, Washington University of Saint Louis MO

Saint Louis MO 63130 USA

Email addresses of authors:

Bin Rao (bin.rao.uc@gmail.com), Ruiying Zhang (ruiying.zhang@wustl.edu), Lei Li (mailto:lei.li@wustl.edu), Jin-Yu Shao (shao@wustl.edu), Lihong V. Wang (lhwang@wustl.edu)

Corresponding author: Lihong V. Wang. Present address: Departments of Medical Engineering and Electrical Engineering, California Institute of Technology, MC 138-78, 1200 E. California Blvd., Pasadena, CA 91125. Phone: (626) 395-1959; Fax: (626) 395-1347; Email: LVW@caltech.edu.

SUPPLEMENTARY INFORMATION

I. Animal preparation

Adult Swiss Webster (Hsd:ND4) mice were used for all *in vivo* imaging experiments. During the surgery, the mouse was first secured using a tooth bar on a platform and maintained under anesthesia with 1.5–2.0% vaporized isoflurane. The scalp was removed, then cyanoacrylate glue was applied to the gap between the back of the mouse's head and an aluminum plate with a "U" shaped dent, as shown in figure 1s. Because the plate was secured to the platform, motions of the mouse head relative to the imaging setup were avoided. A 3 mm by 3 mm cranial window was then created on the mouse skull with dental drill, and the isoflurane level was decreased to ~0.5% after this preparatory surgery. For electrical stimulation experiments, stimulation electrodes were inserted at the edge of the cranial window and fixed on the skull surface with cyanoacrylate glue. For 4-Aminopyridine (4-AP) drug stimulation experiments, direct injection of 4-AP in the neocortex layer within the cranial window was used to induce epilepsy. First, 20 µM of DPA dye was injected into the cranial window, and allowed to stain the cranium for 20 minutes before imaging. After the imaging procedures, the animal was euthanized. All experimental procedures were carried out in conformance with laboratory animal protocols approved by the Animal Studies Committee at Washington University in St. Louis.

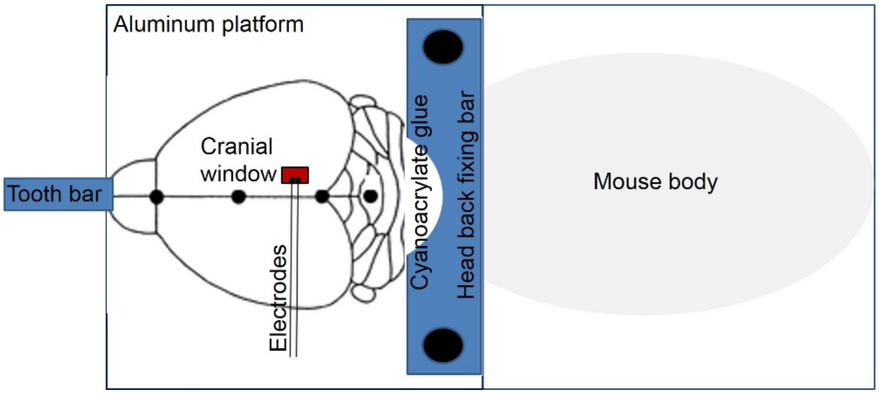

Figure s1. Schematic of animal preparation

II. Experimental setup for *in vitro* photoacoustic cell membrane potential imaging

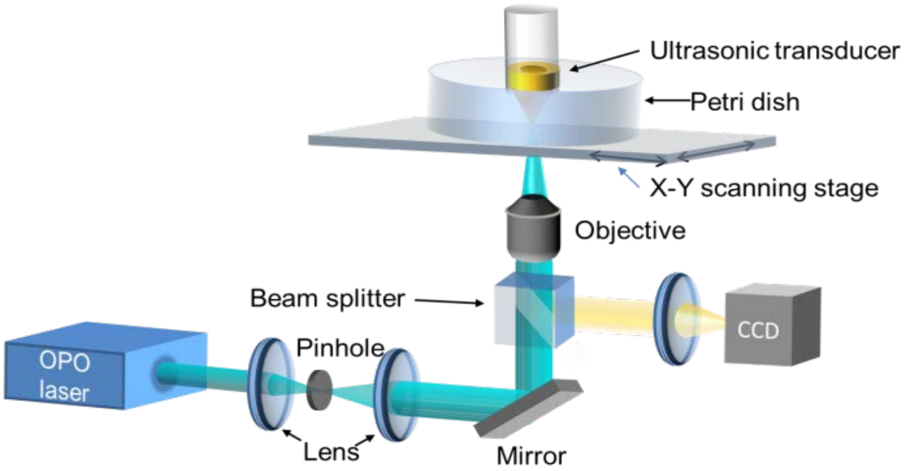

Figure s2. Experimental setup for *in vitro* photoacoustic cell membrane potential imaging

A transmission-mode optical-resolution photoacoustic microscope, as shown in figure s2, was used for *in vitro* voltage imaging of the HEK-293 cell membranes. In figure s2, an integrated diode-pumped Q-switched laser and optical parametric oscillator (OPO) system (NT242-SH, Ekspla) generate laser pulses (5 ns pulse

width, 1 kHz pulse repetition rate) at 488 nm. The laser pulse is expanded and then focused by a 4x objective (Zeiss, 0.1 NA). The focused laser light induces an abrupt temperature rise in an illuminated absorber and excites photoacoustic waves, which are acoustically coupled by deionized water and detected by an ultrasonic transducer. The photoacoustic signal is amplified, digitized, and recorded. By raster scanning the sample with an X-Y scanning stage, a three-dimensional image of the sample is formed. A depth-encoded maximum-amplitude projection (MAP) image of the 3D data set shows the image in 2D format.

III. Experimental setup for *in vivo* photoacoustic mouse brain imaging

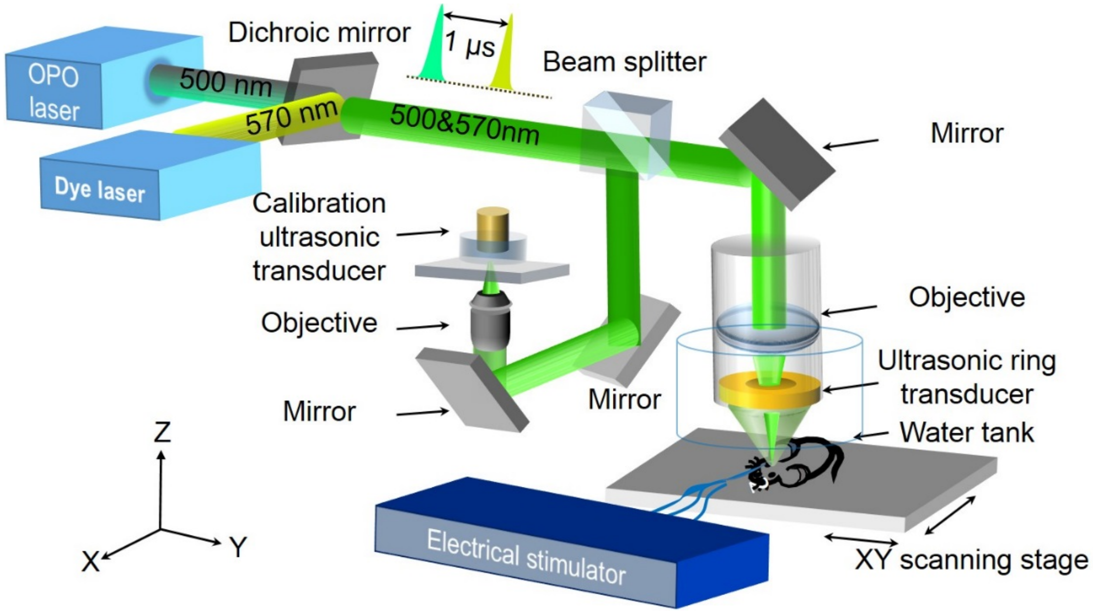

Figure s3. Experimental setup for *in vivo* photoacoustic mouse brain imaging

For *in vivo* mouse brain imaging, we constructed the PAM shown in figure s3. The excitation light sources are (1) an OPO laser system (NT242-SH, Ekspla) and (2)

a dye laser (Credo, Sirah) tuned to 570 nm and pumped by a 532 nm Nd:YLF laser (INNOSLAB, Edgewave). The 570 nm laser pulse is delayed by 1 μs relative to the 500 nm laser pulse. A dichroic mirror combines the 500 nm and 570 nm laser beams. To calibrate the laser pulse energy, a beam splitter splits 10% of the laser energies to a transmission-mode optical-resolution photoacoustic microscope that uses a black tape as a sample. The remaining 90% of the laser energy is delivered to a miniature photoacoustic imaging probe consisting of an achromatic lens (AC064-013-A, Thorlabs) and a customized ring-shaped focused ultrasonic transducer. The mechanical design of the miniature photoacoustic imaging probe allows adjusting the ring transducer for confocal optical and acoustic foci. During imaging, the ring transducer is immersed in the water tank, with a plastic membrane as its bottom. Electrical stimulation electrodes (MX216FW, FHC) are inserted around the edge of the cranial window. Stimulation pulses are sent by an electrical stimulator (Micro-stimu III, World Precision Instruments).

IV. Fluorescence verification of HEK-293 cell membrane voltage response to potassium ion concentration change

As described In the Methods section, four extracellular media with potassium concentrations of 12.7 mM, 61.4 mM, 134.8 mM, and 296.0 mM were prepared by adding KCl to the initial extracellular medium and adjusting the cell membrane resting potentials by 20 mV, 60 mV, 80 mV, and 100 mV, according to equation (7). Although we did not perform quantitative voltage measurements, we sought to use fluorescence imaging to verify that the cell membrane voltages were indeed altered by the extracellular potassium concentrations.

Human embryonic kidney 293 (HEK-293) cells were cultured in DMEM (Invitrogen), supplemented with 10% fetal bovine serum and incubated at 37 °C with 5% CO_2. For photoacoustic microscopy, HEK-293 cells were plated into a 35 mm glass-bottom Petri dish (P35GCOL-0-14-C, MatTek) one day before the experiment. In order to produce a loading concentration of 2 µM di-4-ANEPPS (D1199, Molecule Probes) for fluorescence imaging labeling, a small amount of 2 mM di-4-ANEPPS stock solution in DMSO was added to the HEK-293 cell incubation medium. Pluronic® F-127 (0.05%) was also included in the loading solution to aid the solubility of the dye. After incubation for 10–20 minutes in the presence of the dye, cells were washed three times in dye-free medium. An inverted Olympus IX61 microscope was used for imaging. A baseline fluorescence image corresponding to a 0 mV cell membrane resting potential change was

acquired with the initial extracellular medium. Then the medium was sequentially replaced by extracellular media with potassium concentrations of 12.7 mM, 61.4 mM, 134.8 mM, and 296 mM, and immediately imaged after each replacement.

Figure s4a shows how fluorescence signals changed in response to cell membrane resting potential changes. The larger the HEK-293 cell membrane resting potential changes, the larger the photoacoustic signal changes. Figure s4b quantifies the fractional fluorescence signal changes due to cell membrane voltage changes. Dead cells with significantly brighter intensity are excluded from the calculations for the curve in figure s4b.

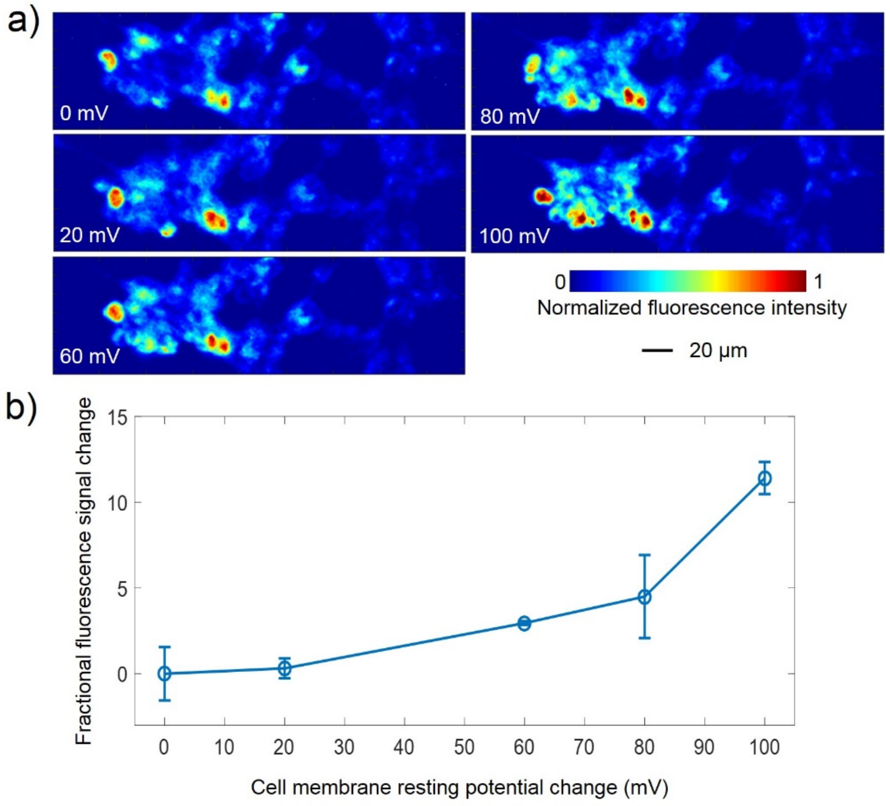

Figure s4 Fluorescence responses to cell membrane resting potential changes

V. Photoacoustic spectroscopy of the DPA-stained HEK-293 cell membrane

at two different membrane potentials

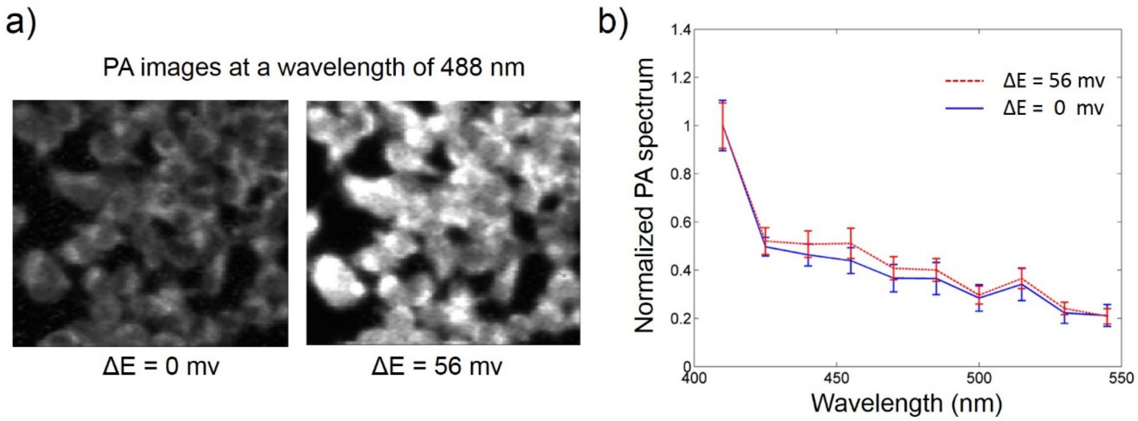

Figure s5 a) HEK-293 PA images at 488nm for 0 mv and 56 mv cell membrane potentials. b) Two normalized PA spectra between 400 – 570 nm.

As mentioned in the manuscript, a voltage-dependent molar optical absorption coefficient (μ) is associated with a voltage-dependent optical absorption spectrum. To investigate whether the voltage-sensitive mechanism is due to μ, we first varied the excitation wavelength of the OPO laser in figure s2 between 400 nm and 570 nm to acquire the PA spectrum of DPA molecules stained on the HEK-293 cell membrane under a 0 mv membrane potential. Next, we acquired the PA spectrum under a 56 mv membrane potential after the external K+ concentration was adjusted. Figure s4a shows PA images of DPA-stained HEK-293 cells illuminated by a 488 nm excitation laser at 0 mv and 56 mv membrane potentials. Figure s4b shows two normalized PA spectra of DPA molecules in the HEK-293 cell membrane between 400 – 570 nm for 0 mv (blue solid line) and 56 mv (red dashed

line) cell membrane potentials. The error bars identify the standard deviation of the measurements. Statistically, we conclude that the normalized PA absorption spectrum remains unchanged when the two different membrane potentials are applied.

VI. Measuring adsorbed DPA molecules with a spectrophotometer

To further prove the DPA voltage contrast mechanism, we performed another experiment based on spectrophotometry instead of photoacoustics. The number of DPA molecules adsorbed on HEK-293 cell membranes equals the total number of DPA molecules minus the number of DPA molecules in the solvent. The number of DPA molecules in the solvent was measured by a spectrophotometer after centrifuging and sampling operations. The measurement results, shown in figure s5, agree with the photoacoustic measurement results. More DPA molecules are adsorbed to cell membranes as the cell membrane voltage change increases.

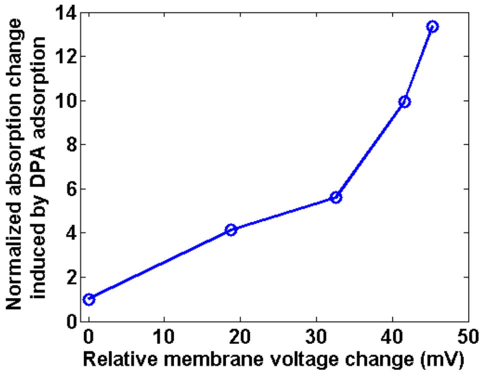

Figure s6. Normalized DPA absorption change versus relative membrane voltage change

VII. Measuring DPA and blood concentrations in DPA and blood mixtures

For *in vivo* experiments, 20 μM DPA was used to stain a mouse brain through a cranial window. However, the effective DPA concentration on the brain surface was significantly higher than 20 μM because of the strong adsorption of DPA by the lipid membrane. To estimate the effective DPA concentration in the lipid membrane, the ratio of the PA signal from 1 mM DPA dye and the PA signal from the lipid membrane of HEK-293 cells stained with 20 μM DPA was measured as 8.3. Thus, the effective DPA concentration of the 20 μM DPA stained lipid membrane was 1 / 8.3 = 0.12 mM. Next, we performed phantom experiments to test our method for separating DPA and hemoglobin signals.

A 20 mM DPA solution was purchased from Biotium Inc. Lysed bovine blood was purchased from QuadFive.com. DPA and blood mixture phantoms were made by mixing varied concentrations of DPA in a diluted lysed blood solution and injecting it into a laboratory tube (CAT. No. 508-001, Dow Corning) with a 0.3 mm inner diameter and 0.64 mm outer diameter. The DPA molar concentrations and blood volume concentrations for the two groups of phantoms are shown in Table 1.

Table 1

**Group 1**	Tube 1	Tube 2	Tube 3	Tube 4	Tube 5
DPA	0 mM	0.1 mM	0.2 mM	0.5 mM	1.0 mM
Blood	10%	10%	10%	10%	10%
**Group 2**	Tube 6	Tube 7	Tube 8	Tube 9	Tube 10
DPA	0.2 mM	0.2 mM	0.2 mM	0.2 mM	0.2 mM
Blood	10%	90%	20%	40%	80%

We performed calibration with two phantoms in each group and plotted both preset points and measured points in 2D concentration-space. Figure s6a shows the measured DPA molar concentrations of the tubes in group 1 versus the preset values. Figure s6b shows the measured volume concentrations of blood in tubes in group 2 versus the preset values. The phantom experimental results prove that our method of separating DPA and blood signals is valid.

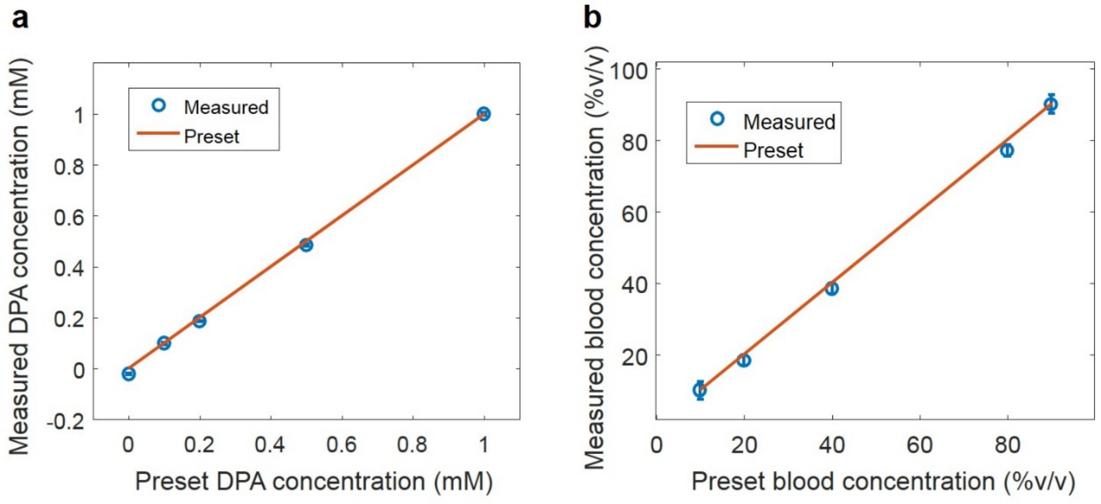

Figure s7. Measured DPA and blood concentrations versus preset values

VIII. Fluorescence verification of *in vivo* mouse brain surface voltage response to electrical stimulation

A fluorescence voltage dye (di-4-ANEPPS) and an upright Olympus BX61W1 microscope were used to verify the *in vivo* mouse brain surface voltage response to electrical stimulation. Animal preparation is detailed in SI, section I. After the creation of a cranial window, the animal brain surface within the cranial window was stained with a 20 µM Di-4-ANEPPS loading solution for 15 minutes. The loading solution contained 0.05% Pluronic® F-127. An area of 136 µm by 153 µm within the red window (1S) in figure s8a was scanned at 12.32 frames per second before and during the electrical stimulation sequence shown in figure 2a. The excitation laser wavelength was 488 nm. The fluorescence signals were integrated for each frame and analyzed in the frequency domain. The top figure of figure s8b shows the baseline signal in the frequency domain, and the bottom figure shows two voltage response peaks at 3.2 Hz and 6.1 Hz in response to the electrical stimulation.

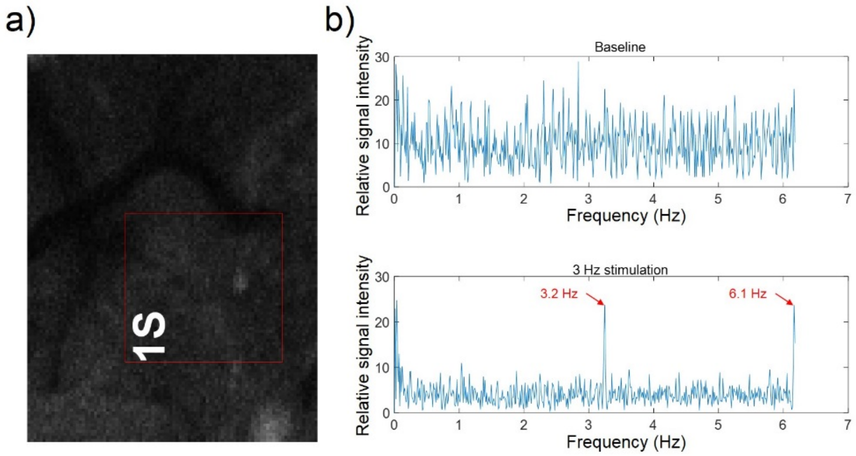

Figure s8. Brain surface fluorescence voltage responses to electrical stimulation

IX. Photoacoustic computed tomography of *in vitro* HEK-293 cell clusters through *ex vivo* thick brain tissue

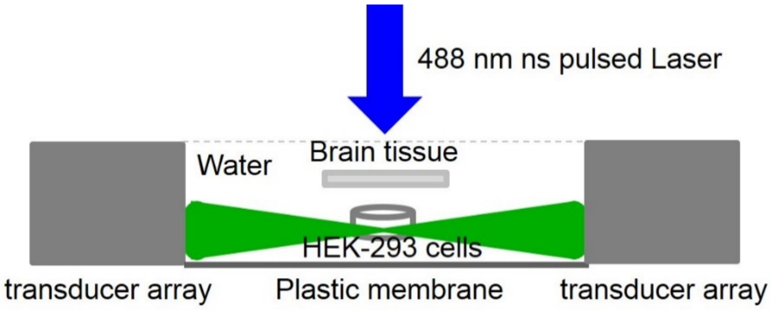

Figure s9. Photoacoustic computed tomography setup for imaging *in vitro* HEK-293 cell clusters through *ex vivo* thick brain tissue

The photoacoustic computed tomography (PACT) setup for imaging *in vitro* HEK-293 cell clusters through *ex vivo* thick brain tissue is shown in figure s9. An OPO laser generated 488 nm nanosecond (ns) pulses (10 ns pulse width, 3 mJ laser pulse energy) for imaging. The collimated laser beam passed through *ex vivo* thick brain tissue before reaching the HEK-293 cell clusters in an agarose tube. Upon

laser excitation, photoacoustic waves generated by the HEK-293 cells (stained with DPA) were detected by a circular transducer array (5 MHz central frequency, 512 elements). A back-projection algorithm was used to reconstruct PACT images. The calculation of the CNR for figure 4a included the following steps:

(1) Define the background:

background = mean (PA amplitudes outside the cell area).

(2) Define the signal from the cell area:

signal = mean ((PA amplitude>= background) within the cell area).

(3) Define the baseline signals from the cell area:

base = mean ((PA amplitude < backgr) within the cell area).

(4) Define the noise as the standard deviation within the background area:

noise = stdev (PA amplitudes outside the cell area).

(5) Finally, calculate the CNR:

CNR = (signal-base) / noise.

X. Calculation error of the voltage response signal

Calculation errors originate from noise in the acquired data. The PA signal at 500 nm has two components, one from Hb and another from DPA dye. The second component is the voltage response signal we want to calculate. We present the following equations from the manuscript:

$$\mu_{Hb500}\mathrm{M}_{Hb} + \mu_{DPA500}\mathrm{M}_{DPA} = \mathrm{p}_{500} \tag{3}$$

$$\mathrm{M}_{DPA} = \left(\mathrm{p}_{570} - \frac{\mu_{Hb570}}{\mu_{Hb500}}\mathrm{p}_{500}\right) / \left(\mu_{DPA570} - \frac{\mu_{Hb570}}{\mu_{Hb500}}\mu_{DPA500}\right) \quad (5)$$

We can calculate the voltage response signal as

$$\mu_{DPA500}\mathrm{M}_{DPA} = \frac{\frac{\mu_{Hb570}}{\mu_{Hb500}}\mathrm{p}_{500} - \mathrm{p}_{570}}{\frac{\mu_{Hb570}}{\mu_{Hb500}}\frac{\mu_{DPA500}}{\mu_{DPA570}} - 1} \times \frac{\mu_{DPA500}}{\mu_{DPA570}}$$

From the known absorption curves of DPA and Hb, we calculated $\frac{\mu_{Hb570}}{\mu_{Hb500}}$ as 2.143 and $\frac{\mu_{DPA500}}{\mu_{DPA570}}$ as 19.8.

Thus,

$$\mu_{DPA500}\mathrm{M}_{DPA} = 1.024 \times \mathrm{p}_{500} - 0.478 \times \mathrm{p}_{570}$$

So the error of the voltage response signal $Error(\mu_{DPA500}\mathrm{M}_{DPA})$ is

$$Error(\mu_{DPA500}\mathrm{M}_{DPA}) = 1.024 \times Error(\mathrm{p}_{500}) - 0.478 \times Error(\mathrm{p}_{570})$$

$$|Error(\mu_{DPA500}\mathrm{M}_{DPA})| \leq 1.5 \times |Error(\mathrm{p})|$$

Thus, the absolute calculation error of the voltage response signal is less than 1.5 times the noise of the PA imaging system.

XI. Estimation of hemoglobin and DPA contributions to PA signal

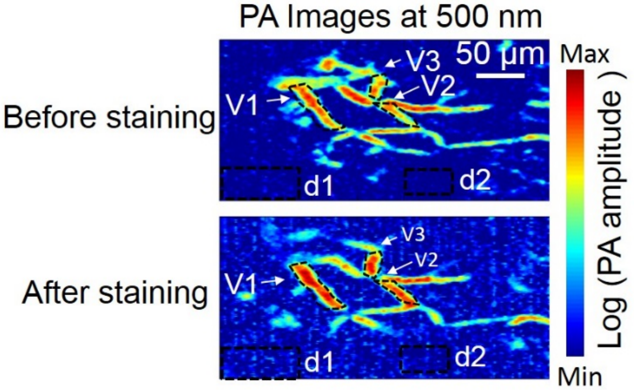

Mean PA signals (a.u.)	d1	d2	v1	v2	v3
PA (Hb)	603	557	2469	2171	2652
PA(Hb)+PA(DPA)	730	677	3089	2826	3053
PA (DPA)	127	120	620	655	401
PA(Hb) / PA(DPA)	4.7	4.6	4.0	3.3	6.6

Figure s10 Effects of DPA staining on mouse cranial window

Figure s10 compares brain images at 500 nm before and after 20 µM DPA dye staining. The PA signal change due to DPA dye staining is obvious because of the strong lipid membrane adsorption effect. The mean PA signal before staining, from region d1, is dominantly from capillary blood vessels. For region d1, the mean PA signal after DPA staining is dominantly from capillary blood vessels and adsorbed DPA dye. Thus, the dye signal at 500 nm is 730-603 = 127. We can estimate the ratio of the hemoglobin signal to the DPA signal within region d1 as

$$\frac{PA_{Hb500}}{PA_{DPA500}} = 603/127 \;\; = \;\; 4.7 \;\; .$$

We can estimate the ratio at 570 nm from the known absorption curves of Hb and DPA:

$$\frac{PA_{Hb570}}{PA_{DPA570}} = \frac{PA_{Hb570}}{PA_{Hb500}} \times \frac{PA_{Hb500}}{PA_{DPA500}} \times \frac{PA_{DPA500}}{PA_{DPA570}}$$

$$\frac{PA_{Hb570}}{PA_{DPA570}} = \frac{\mu_{a_{Hb570}}}{\mu_{a_{Hb500}}} \times \frac{PA_{Hb500}}{PA_{DPA500}} \times \frac{\mu_{a_{DPA500}}}{\mu_{a_{DPA570}}} = 2.143 * 4.7 * 19.8 = 200$$

At 570 nm, the hemoglobin signal is dominant. However, at 500 nm, the DPA dye contribution is significant. In other labeled areas of the figure s8, the ratio of $\frac{PA_{Hb500}}{PA_{DPA500}}$ at 500 nm varies from 3.3 to 6.6.

XII. Frequency components of the electrical stimulation pulse train

We assume the single pulse electrical stimulation function is written as

$$g(t) = \begin{cases} A, & |t| \leq \frac{T}{2} \\ 0, & |t| > \frac{T}{2}. \end{cases}$$

The Fourier-transformation of $g(t)$ is the frequency-domain signal:

$$G(f) = AT\frac{\sin(\pi f T)}{\pi f T} \quad .$$

The electrical stimulation pulse train is written as the convolution of $g(t)$ with a comb function:

$$h(t) = g(t) * comb(t)$$

$$comb(t) = \sum_{n=-\infty}^{+\infty} \delta(t - nT')$$

$$* \quad convolution.$$

Here T is 300 µs, and T' is 333 ms, so $\frac{1}{T'}$ is 3 Hz.

The Fourier-transformation of the pulse train h(t) is

$$H(f) = G(f)\ \frac{1}{T'} \quad \sum_{k=-\infty}^{+\infty} \delta\left(f - k\frac{1}{T'}\right)$$

$$H(f) = AT\frac{\sin(\pi f T)}{\pi f T}\ \frac{1}{T'} \quad \sum_{k=-\infty}^{+\infty} \delta\left(f - k\frac{1}{T'}\right)$$

Thus, the pulse train stimulation has frequency components only at $k\frac{1}{T'}$, which is an integer multiple of 3 Hz.
